# New approach methodologies (NAMs) to support regulatory assessment of developmental immunotoxicity – a new PARC project

**DOI:** 10.3389/ftox.2026.1740390

**Published:** 2026-04-17

**Authors:** Nicola M. Smith, Véronique M. P. de Bruijn, Rob J. Vandebriel, Rita Hargitai, Katalin Lumniczky, Aafke W. F. Janssen, Karsten Beekmann, Birol Usta, Julia Tigges, Christiane Spruck, Selma Hurem, Martina Iulini, Emanuela Corsini, Anne Bado-Nilles, Rémy Beaudouin, Ioanna Katsiadaki, Irene Cano, Marion Sebire, Humblenoble Stembridge Ayuk, Violeta Stojanovska, Ana C. Zenclussen, Etienne B. Blanc, Saadia Kerdine-Römer, Rafaela Lacerda, Luísa Romão, Pablo Monfort-Lanzas, Johanna M. Gostner, Archibold Mposhi, Jonathan D. Turner, Giorgia Del Favero, Birgitte Lindeman

**Affiliations:** 1 Department of Chemical toxicology, Norwegian Institute of Public Health, Oslo, Norway; 2 Centre for Health Protection, National Institute for Public Health and the Environment (RIVM), Bilthoven, Netherlands; 3 Unit of Radiation Medicine, Department of Radiobiology and Radiohygiene, National Centre for Public Health and Pharmacy, Budapest, Hungary; 4 Wageningen Food Safety Research (WFSR), Wageningen University and Research, Wageningen, Netherlands; 5 IUF – Leibniz Research Institute for Environmental Medicine, Dusseldorf, Germany; 6 Faculty of Veterinary Medicine, Norwegian University of Life Sciences, Aas, Norway; 7 Laboratory of Toxicology and Risk Assessment, Department of Pharmacological and Biomolecular Sciences “Rodolfo Paoletti”, Università degli Studi di Milano, Milan, Italy; 8 Institut national de l’environnement industriel et des risques, Verneuil-enHalatte, France; 9 Centre for Environment, Fisheries and Aquaculture Science (Cefas), Weymouth, United Kingdom; 10 Department of Environmental Immunology, Helmholtz Center for Environmental Research- UFZ, Leipzig, Germany; 11 German Center for Child and Adolescent Health (DZKJ), Leipzig/Dresden, Leipzig, Germany; 12 Perinatal Immunology Research Group, Medical Faculty, Saxonian Incubator for Clinical Translation (SIKT), University of Leipzig, Leipzig, Germany; 13 Université Paris Cité, INSERM, Health & Functional Exposomics - HealthFex, Paris, France; 14 Université Paris-Saclay, Inserm, Inflammation, Microbiome & Immunosurveillance, Orsay, France; 15 Department of Human Genetics, National Institute of Health Dr Ricardo Jorge, Lisboa, Portugal; 16 BioISI — Biosystems & Integrative Sciences Institute, University of Lisbon, Faculty of Sciences, Campo Grande, Lisbon, Portugal; 17 Institute of Medical Biochemistry, Biocenter, Medical University of Innsbruck, Innsbruck, Austria; 18 Institute of Bioinformatics, Biocenter, Medical University of Innsbruck, Innsbruck, Austria; 19 Department of Infection and Immunity, Luxembourg Institute of Health, Esch-sur-Alzette, Luxembourg; 20 Institute of Food Chemistry and Toxicology, Faculty of Chemistry, University of Vienna, Vienna, Austria

**Keywords:** developmental immunotoxicity, environmental chemicals, hazard assessment, new approach methodologies, PARC, regulatory science

## Abstract

The current testing strategy for the assessment of developmental immunotoxicity (DIT) in European chemicals regulations has well recognised limitations. In response to these limitations, the Partnership for the Assessment of Risks from Chemicals (PARC) initiated a 4-year DIT project in May 2025. This project comprises 14 partner institutions and will tackle two primary objectives: a) to enhance the DIT knowledgebase and refine the understanding of critical phases of immune system development, and b) to facilitate the transition towards the use of New Approach Methodologies (NAMs) in DIT risk assessment. The first objective will be approached by reviews of existing literature and the continued advancement of a physiological map of human immune system development. The reviews and the physiological map will in turn serve as foundational tools to support NAMs and Adverse Outcome Pathway (AOP) development. Addressing the second objective, the project has a short-term aim to promote a testing strategy that leverages current immunotoxicity assays and their modifications to inform regulatory decision processes such as screening, prioritisation and read-across analyses. In the longer term, novel NAMs, encompassing developmental processes, will be developed and assessed for their regulatory applicability. While the primary focus of this PARC project is on the enhancement of human DIT risk assessment, it also aims to contribute to the advancement of ecotoxicological evaluation of immunotoxicity.

## 1 Introduction

Developmental immunotoxicity (DIT) is defined as adverse effects on the immune system resulting from exposure to environmental risk factors prior to adulthood, including chemical, biological, physical, or physiological factors (DeWitt et al., 2012).

The developing immune system is inherently sensitive to environmental factors (Dietert, 2014; Luebke et al., 2006a), including viruses, bacteria, drugs, environmental chemicals and stress, resulting in a significant degree of heterogeneity that confers variations in susceptibilities to disease. Environmental factors, including air pollution, lead, polychlorinated biphenyls (PCBs) and per- and polyfluoroalkyl substances (PFAS) have been associated with impaired immune system functioning and disruption of immune system development leading to increased risk of, e.g., allergies, autoimmune diseases, infection or increased disease severity (Hessel et al., 2015), as well as reduced vaccine responses in children (Ehrlich et al., 2023).

In EU chemical regulations, the current approaches are considered to lack sensitivity for the identification of substances with developmental immunotoxic modes of action. This is partly due to the limited assessment of changes in immune system function and of exposures during critical developmental periods. The T-cell dependent antibody response (TDAR) assay included in the extended one-generation reproductive toxicity study in rodents (OECD TG 443, EOGRTS) (OECD, 2025) assesses developmental exposure and is the only functional immunotoxicity endpoint included in the test requirements. Of note, the DIT cohort (Cohort 3) is only included in the EOGRTS if triggered by a concern for immunotoxicity. However, there is a general lack of accepted sensitive triggers, and few chemicals have so far been tested for their potential to cause DIT (Vandebriel et al., 2024).

These regulatory concerns are highlighted in the EU Chemicals Strategy for Sustainability and by ECHA in their KARC (Key Areas of Regulatory Challenge) documents, published yearly from 2023, in which they identify DIT as a regulatory need and a prioritized area for further research (ECHA, 2025). Importantly, this knowledge gap extends beyond DIT, as chemical-induced immunotoxicity across all life stages remains insufficiently characterized, and current testing approaches are unable to detect the full spectrum of immune-related adverse outcomes. The potential immunotoxic effects of chemicals are currently not considered in ecotoxicological risk assessments, even though it has been shown that, e.g., endocrine-related immunotoxicity may take place at exposure concentrations below systemic toxicity (Segner et al., 2013).

The evaluation of immunotoxicity makes limited use of the considerable general knowledge and existing non-animal tests and alternative vertebrate bioassays. However, the recent OECD DRP (No 360: *In Vitro* Tests Addressing Immunotoxicity With a Focus on Immunosuppression (OECD, 2022) and the publication “*Consensus on the Key Characteristics of Immunotoxic Agents as a Basis for Hazard Identification*” (Germolec et al., 2022), provide a foundation for developing alternative test strategies for identifying compounds that are likely to show adult immunotoxicity. In addition, concerted efforts are being made to close existing knowledge and testing gaps in chemical-induced adult immunotoxicity, including the ongoing PARC (Hargitai et al., 2024) and OECD initiatives focused on improving assessment strategies for respiratory sensitizers.

To support the assessment specifically of DIT, there is a need to better define the critical windows of immune system development. The current knowledge on the foetal immune system is to a large degree based on information from animal models. However, due to recent technological advances these data are now complemented by detailed characterisation of human prenatal immune cells (Suo et al., 2022; Haniffa et al., 2025) that together with advancements in stem cell-based models greatly benefit human-centric *in vitro* assay development.

**FIGURE 1:**
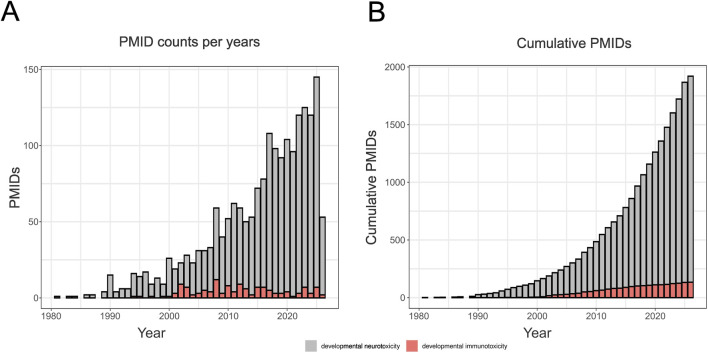
Publication trends related to DIT. **(A)** Annual number of PubMed-indexed articles returned by the queries “developmental immunotoxicity” [All Fields] and “developmental neurotoxicity” [All Fields]. **(B)** Cumulative number of articles indexed by PubMed for both terms over the same period. The search was performed in November 2025 using the easyPubmed library in R.

The focus of the PARC NAMs project will be on human health. However, some of the approaches will also be applicable in ecotoxicology. As can be seen from Figure 1, there has been a steady flow of publications on DIT since 2000, with no more than 12 publications per year and a total of 134 publications identified up to 2026. This highlights the underrepresented nature of this toxicological endpoint, given its importance to long-term health. For comparison, the literature on developmental neurotoxicity (DNT) is substantially larger, with 1,786 publications identified up to 2026 and a peak of 138 publications in 2025. This contrast further emphasizes the relative scarcity of DIT-focused studies while also demonstrating that a consistent body of literature exists to support the proposed project goals.

### 1.1 PARC DIT NAMs project overview

This publication introduces a new PARC (Partnership for the Assessment of Risks from Chemicals) project addressing DIT and New Approach Methodologies (NAMs) development which was launched in May 2025.

The first step of the PARC DIT NAMs project (Subproject 1) addresses current knowledge gaps related to chemical exposure and modulation of the developing immune system. More specifically, the project will update the knowledge base for DIT by identifying critical windows of immune system development and further advancing a physiological map of the human developing immune system.

The second step of the project (Subproject 2A-C) aims to; a) assess existing immunotoxicity assays and their adaptations to explore their use in a regulatory DIT context, including supporting screening, prioritisation and read-across processes, and b) to develop novel NAMs using a variety of models to evaluate the impact of chemicals on the developing immune system.

This NAMs project is closely related to the ongoing immunotoxicity, NAM and Adverse Outcome Pathway (AOP) development activities in PARC (WP5), which cover adult immunotoxicity. However, testing for DIT has additional complexity and challenges, and currently lacks well-characterized non-animal methods.

**FIGURE 2:**
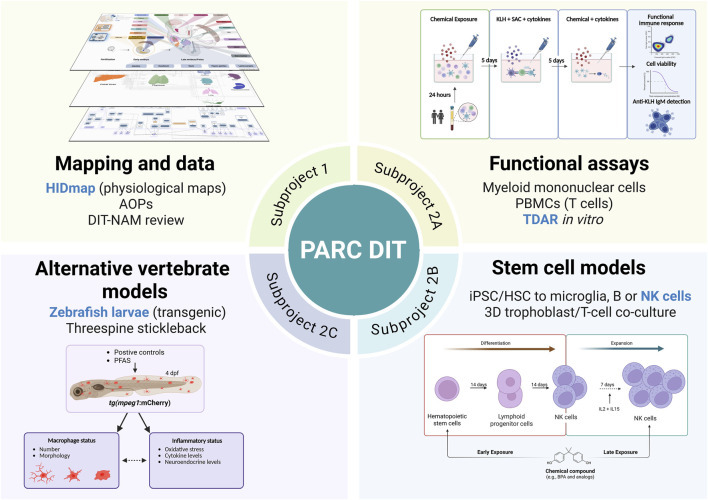
Project outline. The project is divided into two closely related subprojects. The non-lab activities in subproject one reviews and structures the current knowledge of DIT and on immune cell development whilst subproject 2A-C focuses on developing NAMs to support the evaluation of DIT. Figure created in BioRender.

The 4-year project described here (Figure 2) aims at strengthening the regulatory assessment of potential DIT chemicals by facilitating the transition towards the use of NAMs in the DIT risk assessment strategy. The approach is human-centric by providing NAMs that are human-relevant and fit-for-purpose, and also aims to reduce the reliance on animal testing aligning with the 3Rs principle (Replacement, Reduction, and Refinement) (The Principles of Humane Experimental Technique, 1960). Collaborative efforts with other immunotoxicity projects, both within and outside of PARC, will ensure that the developed tests are robust and aligned with the latest scientific advancements. The longer-term goal is to develop a comprehensive *in vitro* testing strategy to evaluate the immunotoxic potential of compounds that may interfere with immune system development, thereby improving regulatory frameworks and enhancing the protection of vulnerable populations.

**FIGURE 3:**
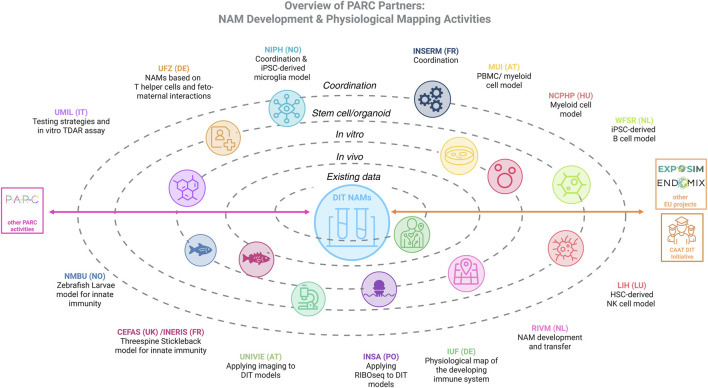
Overview of project partners and main activities. Interactions with other PARC, EU and internation projects are indicated by arrows. Figure created in BioRender.

An overview of the PARC DIT NAMs project partners and interactions with other PARC projects and non-PARC initiatives are shown in Figure 3.

### 1.2 Preparatory workshop

A workshop entitled “*Advancing Developmental Immunotoxicity Testing in PARC*” was held in March 2024 to bring together academics, regulators, PARC partners, and other stakeholders with the aim of strengthening collaboration in the development and regulatory application of non-animal approaches for chemical risk assessment.

Day 1 (1 March 2024) addressed the effects of chemicals on immune system development, regulatory needs, and the role of NAMs in assessing DIT. On the second day (4 March 2024) the focus was on available and emerging NAMs, including proposals for the development of a PARC DIT project. The full agenda and list of speakers are available in Supplementary Table S1.

Through expert presentations, discussions, and breakout sessions, participants identified critical research and regulatory gaps as well as opportunities for methodological refinement. Potential synergies with related international initiatives such as the International Working Group on Alternatives to *in vivo* DIT Testing led by the Johns Hopkins University Center for Alternatives to Animal Testing (CAAT) were also discussed. The workshop outcomes laid the foundation for PARC’s continued commitment to advancing innovative, animal-free strategies for assessing DIT and supporting their integration into next-generation risk assessment frameworks. More detailed brainstorming on the challenging process of driving a collective effort across numerous institutions to achieve a common goal was conducted in two 45-min breakout sessions. The structured discussions and guiding questions are described in detail in Supplementary Table S2.

Key takeaways and consensus on the next steps for PARC partners and stakeholders were collected, and from these discussions a project description of the PARC work presented in the following sections was developed.

## 2 Update of DIT knowledge base and further development of the HIDmap (subproject 1)

The main project activities are shown in Figure 2. Subproject one undertakes a comprehensive review of existing data on DIT and on the ontogeny of the immune system. It applies the concept of physiological maps and the AOP framework to structure these data, thus facilitating NAM development and supporting regulatory processes. Although a priority initiative for the first year, activities under Subproject one will proceed in parallel with the experimental activities outlined in subproject 2. One of the objectives of the PARC DIT NAMs project is the preparation of a comprehensive review of NAMs in DIT testing. This review will provide an updated overview of the available data to advance our understanding of how early-life exposure to environmental chemicals can interfere with immune system development. It will summarize key findings on sensitive windows of immune vulnerability and highlight recent progress in human-relevant models, including stem cell-based systems, organoids, organ-on-chip platforms, and alternative non-human vertebrate models. By integrating mechanistic insights and methodological advances, the review aims to support the improvement and expansion of DIT testing frameworks and the development of predictive tools for regulatory and research applications.

A key challenge in the regulatory assessment of DIT is the limited availability and oversight of mechanistic, human-relevant reference knowledge. To address this, the Human Immune System Development Map (HIDmap) has been developed by the IUF - Leibniz Research Institute for Environmental Medicine as an expert-curated, machine-readable, and publicly accessible systems-level resource^1^.

The HIDmap provides the first visual and structured representation of prenatal human immune system development. In its current version, it integrates data from 21 peer-reviewed studies into a modular, organ- and process-specific network, reflecting hematopoiesis, immune cell differentiation, migration, and tissue-specific maturation for the developmental window of *in utero* development. Hosted on the MINERVA platform using Systems Biology Graphical Notation (SBGN) (Gawron et al., 2016), it enables both interactive exploration and standardized data sharing. The map is designed to support researchers and regulators in anchoring NAMs to key events (KEs) relevant to DIT.

Within the PARC DIT NAMs project, the aim will be to advance and validate the HIDmap content by applying several coordinated efforts. A structured literature search is ongoing to identify new and relevant developmental immunology data. In parallel, the inclusion of information on monogenic immune disorders available via the curated database from the International Union of Immunological Societies Expert Committee will be integrated (Poli et al., 2025).

The HIDmap also serves as a foundational tool for the development of AOPs, and, by providing a transparent, human-centric mechanistic framework, it addresses one of the key regulatory challenges identified by ECHA (ECHA, 2025); the need for validated and biologically plausible NAMs for DIT assessment.

The physiological map will serve as a new method to support the proposed paradigm shift away from the exclusive use of animal testing to the application of human-relevant approaches by modeling critical developmental steps *in vitro*. It will synthesize the spatial and to some extent also the temporal dynamics of human immune system development, serving as a tool to identify critical processes for assay development and knowledge gaps for targeted research. Importantly, physiological maps are living documents that can be adapted when new data are available and allow contribution also from non-PARC research initiatives.

Complementing the DIT review and HIDmap efforts, a literature search-based approach using PubMed and AOP-helpFinder tool (Jaylet et al., 2025) will be used to identify and extract associations between stressor and event, and between event and event, extending the approach recently applied to refine the AOP for respiratory sensitization (Hargitai et al., 2024). For this, a curated list of DIT reference chemicals identified by the International Working Group on “Alternatives to *in vivo* Developmental Immunotoxicity Testing” led by CAAT will serve as input.

This combined strategy ensures that the mapping of human immune system development and sensitive windows of development is grounded in both mechanistic understanding and chemical relevance.

An additional literature project focuses on comprehensive molecular profiling approaches to delineate their applicability in developmental immunotoxicology. Emerging methods, such as ribosome profiling (Ribo-seq), may offer added value by capturing a functional, translational layer beyond transcript abundance (Eastman et al., 2018). Integrating analyses like Ribo-seq into the already existing developmental models may enhance hazard identification and help understand mechanisms of action for immunotoxic effects at different life stages (Luebke et al., 2006b; Ingolia et al., 2012; Piccirillo et al., 2014). This integration aims to demonstrate how Ribo-seq can be utilized to; a) analyze how hazardous chemicals affect the translatome during gestation, infancy, and adulthood; b) identify potential biomarkers for inflammation-related cancer processes; and c) aid in hazard assessment and biomarker discovery.

## 3 Existing assays, novel NAMs and their potential use in regulatory DIT testing (subproject 2)

Currently, DIT is assessed by cohort three of the extended one-generation reproductive toxicity study (OECD TG 443) (OECD, 2025), which is normally performed in rats. This study is intensive in terms of animal use, time and resources, and the DIT cohort is included only if concern for immunotoxicity is triggered. Therefore, development of an alternative testing strategy is needed that targets processes particularly sensitive to modulation by chemicals during immune system development. The short-term goal is to propose a combination of existing, well-characterized assays that are informative for regulatory decisions like screening, prioritisation, and read-across and as DIT cohort three triggers. An *in vitro* TDAR assay (Iulini et al., 2025) is an example of a potential trigger. In the longer term, the project will contribute to the development of an *in vitro* testing strategy to assess developmental immunotoxicity. To achieve this, adaptation of existing tests and development of novel HSC- and iPSC-based models as well as alternative vertebrate models will be explored. Collaboration with other immunotoxicity projects within and outside of PARC relevant to DIT is and will remain integral to this effort, as it enhances the effectiveness and applicability of the project’s outcomes.

### 3.1 Existing assays and models of immune cell maturation (subproject 2A)

Within the PARC NAMs activities, specific efforts are dedicated to the development and optimization of *in vitro* methods aimed at identifying and characterizing compounds with potential developmental immunotoxic effects. Complementary approaches are being pursued to address both adaptive and innate immune endpoints.

Part of the approach builds on methods that are already optimized or under development within PARC, aiming to identify reliable triggers for DIT. Among the key components, an *in vitro* TDAR assay will be adapted to capture alterations in adaptive immune function following chemical exposure. Such assays have been increasingly explored as human-relevant approaches to assess functional immune responses and provide mechanistic insight into immunotoxicity, supporting their feasibility for screening applications. The ultimate goal is to establish a robust and mechanistically informative testing framework that can serve as an early screening tool to flag potential adult and developmental immunotoxicants in a human-relevant context.

Several project activities target optimization of *in vitro* methods to investigate myeloid cell differentiation using the human promyelocytic cell line THP-1 as a model system (Tsuchiya et al., 1980). The approaches focus on characterizing key molecular and functional markers of monocyte-to-macrophage maturation and their modulation upon exposure to immunotoxic compounds (Noronha et al., 2020). By refining culture conditions, differentiation protocols, and endpoint analyses, the methods aim to improve sensitivity and reproducibility. At the same time, the use of cell lines requires careful consideration, due to biological variability arising, e.g., from ongoing clonal evolution during *in vitro* propagation (Noronha et al., 2020; Ben-Dav et al., 2018). These factors highlight the importance of standardized culture conditions and robust response assays to enable reliable interlaboratory comparisons.

Capitalizing on the technical expertise on the immunofluorescence analysis of crucial mediators of the immune response (Del Favero et al., 2020; Gerner et al., 2020; Hohagen et al., 2024), the applicability of these workflows will be further investigated for the study of the hormonal dependence on the maturation/activation of THP-1 cells (i.e., expression of Toll-like Receptors, elements of the NF-κB pathway). This could support exploring a possible correlation between hormonal status and immunocompetence and be instrumental to the screening of substances active on the endocrine system with respect to potential (developmental) immunotoxicity.

Dendritic cells (DC) play a vital role in the establishment of adaptive immunity as antigen-presenting cells, and alterations in DC function can increase the risk for infections and the development of allergies (Humeniuk et al., 2017). The project will investigate the activation and maturation capacity, epigenomic and transcriptomic alterations of chemical-exposed monocytic cells during monocytic to DC maturation and their ability to alter the balance between Th1 and Th2 immune response (Mainali et al., 2005). Apart from the broadly applied THP-1 cell line, alternative human myeloid progenitor cell lines will also be investigated as possible models for testing immunotoxicants (dos Santos et al., 2009). Peripheral blood mononuclear cells (PBMCs) are a readily available source of primary human immune cells that will be used in the PARC DIT NAMs project. PBMCs enable studies of physiological immune crosstalk and allow for the isolation of specific subsets of immune cells for detailed analysis; however, this comes with increased experimental complexity and donor-to-donor variability.

For adequate host defense and immune homeostasis, the differentiation of naïve CD4^+^ T helper (Th) cells into distinct effector subsets, such as Th1, Th2, Th17, or regulatory Treg cells, is crucial for maintaining a balance between pro-inflammatory and immunoregulatory responses (Zhou et al., 2009). Several studies have shown that disruption of this differentiation equilibrium can lead to a spectrum of disorders, including autoimmune diseases and allergies (Jäger and Kuchroo, 2010). For example, endocrine-disrupting chemicals such as alkylphenols have been reported to differentially modulate CD4^+^ T cells towards a Th2 profile and suppress Th1 functions (Iwata et al., 2004). Moreover, a combined exposure of naïve T cells to bisphenol A (BPA) and bisphenol 3 (BP-3) induced Th17 cell differentiation (Fischer et al., 2024). The experiments will build upon this foundation to develop new methodologies for profiling the effects of environmental chemicals, either as single substances or mixtures, on the differentiation properties of naïve T cells. Due to some nuances of interspecies differences in immune cell signaling between animal models and humans (Skaggs et al., 2019; NAS, 1992; Tuomela et al., 2016; Bjornson-Hooper et al., 2022), it is planned to use NAMs that mimic human physiological interactions between chemicals and immune cells. To achieve this, human PBMCs from healthy female and male donors will be isolated and sorted for naïve CD4^+^ T cells using fluorescence-activated cell sorting (FACS). Human naïve CD4^+^ T cells provide a well-established model to study cytokine-driven helper T cell lineage commitment and allow controlled investigation of Th1, Th2, Th17, and Treg differentiation pathways under defined conditions (Schmitt and Ueno, 2015). Purified naïve CD4^+^ T cells will be exposed to single chemicals or mixtures at real-life concentrations, and the phenotypic differentiation, activation and transcriptional profile of the CD4^+^ T cells will be analyzed using advanced multiparameter flow cytometry (spectral flow cytometry) and quantitative PCR.

Sustaining maternal immune balance while supporting fetal development requires tightly coordinated immune and metabolic processes. A central pathway in this context is the catabolism of the essential amino acid tryptophan via induction of the immunoregulatory enzyme indoleamine 2,3-dioxygenase 1 (IDO-1). Initially, tryptophan depletion was proposed to prevent rejection of the allogeneic fetus by suppressing T-cell responses (Munn et al., 1998). This concept has since been refined, and current understanding emphasizes the dual importance of tryptophan availability for protein synthesis and the roles of kynurenine pathway metabolites in neuronal protection and development, immunomodulation, and as precursor of the essential cofactor nicotinamide adenine dinucleotide (NAD) (Badawy, 2015). In PARC, a PBMC assay using tryptophan metabolism as readout for immunomodulation is currently in use (Gostner et al., 2015), with minor modifications, for characterizing effects of bisphenols on immunometabolism. This assay will be slightly adapted for the purpose of DIT testing by the optional inclusion of additional readouts targeting further immune regulatory pathways and related readouts (e.g., Th1/Th2 polarization and innate inflammatory responses).

### 3.2 Advanced *in vitro* models of human immune cell development (subproject 2B)

Based on the DIT review and the physiological map (HIDmap) activities described in Section 2, we will select critical immunological mechanisms as primary targets for further investigation. *In vitro* methods will be developed to study chemical-induced alterations in these mechanisms. The use of hematopoietic stem cells (HSCs) and iPSCs, which can be differentiated into various immune cell types to model developmental processes and responses to chemicals will be harnessed. Setting up protocols for the differentiation of HSCs and iPSCs into immune cells aims to identify biomarkers for (developmental) immunotoxicity. This approach enables the screening of substances for adverse effects at early hematopoietic stages, that potentially may impact multiple lineages, as well as on more differentiated downstream immune cell types.

Several stem cell-based models for innate and adaptive immune cells that are either under development or are selected for feasibility studies in the second year of the project are described below. Additional stem cell-based models or immunological mechanisms will be selected based on the information gained from Subproject 1.

NAMs based on Natural Killer (NK) cells derived from HSCs will be developed. This includes assessing the functioning (suppressed or stimulated) of derived NK cells. Further identification of pathways/mechanisms that are involved in altered differentiation of HSC to NK cells and mechanisms that underly immunosuppression and immunostimulation will be pursued. The NK model will be tested by exposure to selected BPA analogs at human-relevant concentrations.

Tissue resident macrophages originate from yolk sac progenitor cells (Bian et al., 2020) and are the main resident macrophages of the central nervous system (CNS). Microglia play vital roles in brain development, homeostasis and immune surveillance (Anderson and Vetter, 2019; Kettenmann et al., 2013). *In vitro* culture of microglia has been hampered by the loss of CNS-specific features (Warden et al., 2023; Gosseli et al., 2017). Recently, iPSC-derived models have been produced that more closely resemble the *in vivo* microglia (Warden et al., 2023; Teo et al., 2024), some of these are engineered to express microglial specific transcription factors to direct the differentiation process (Dräger et al., 2022; Chen et al., 2021). The applicability of such models for the development of NAMs to assess the impact of chemical exposure on microglial developmental toxicity will be investigated.

There will be further efforts to develop NAMs that can be used to screen substances for DIT, with a particular focus on B lymphocytes. To achieve this, an *in vitro* method will be established in which iPSCs are differentiated into immune cells. This differentiation process involves several stages, starting with mesoderm specification, followed by the formation of HSCs, and subsequent development into downstream immune cells through either the myeloid or lymphoid lineage.

Various approaches for differentiating iPSCs into HSCs have been reported, including embryoid body formation, co-culture with mouse stromal cells, and feeder-free systems such as the monolayer approach (Tursky et al., 2020; Zheng et al., 2023; French et al., 2015). Given the high variability and low efficiency associated with embryoid body formation, and the reliance on animal material and undefined secreted factors in co-culture with mouse stromal cells, there will be a focus on the development of feeder-free, chemically defined differentiation methods (Iriguchi et al., 2021; Cao et al., 2020). A challenge, however, is the absence of established feeder-free, chemically defined protocols for differentiating iPSCs into mature B lymphocytes.

Early and throughout pregnancy, coordinated communication between maternal immune cells and trophoblasts, the placenta’s key functional cells, ensures proper implantation and fetal growth. Studies, in both animal models and humans, have shown that dysregulation of maternal immune adaptation can lead to pregnancy complications (García-Morales et al., 2026). Previous studies have shown that trophoblast spheroids can be used to study key placental functions, such as proliferation, invasion, and hormone production (Stojanovska et al., 2022). Furthermore, it has been shown, that *in vitro* co-culture of PBMCs from healthy females, activated with SARS-CoV-2 spike peptide does not alter the functionality of trophoblast spheroids (Ayuk et al., 2025). Recently, using a 3D trophoblast model, it was demonstrated that real-life PFAS mixtures can impair trophoblast spheroid function (Xia et al., 2025). To investigate the effects of chemicals on the foetal-maternal crosstalk, work in the project will co-culture a well-established 3D trophoblast spheroid model with naive CD4^+^ T cells isolated from PBMC obtained from peripheral whole blood samples of females within reproductive age. The trophoblast spheroid, and the naive CD4^+^ T cells will first be pre-exposed to the pre-selected chemicals before co-culture and after several days of co-culture, multiple parameters will be assessed, including functional responses (viability, growth, invasion and hormone production) and transcriptomics profiles (migration, apoptosis and proliferation).

### 3.3 Alternative vertebrate models (subproject 2C)

A major challenge in assessing chemical-related immunomodulation is the diversity of potential targets and effects that can occur in exposed organisms (Rehberger et al., 2017). As essential immune system features are evolutionarily conserved (Blom and Ottaviani, 2017), the use of non-protected forms of aquatic vertebrates, such as fish eleuthero embryos offer an alternative platform that can serve as a basis for development of NAMs to support human DIT assessment as well as fill knowledge and regulatory gaps for aquatic vertebrates. Two different models will be used: transgenic zebrafish larvae (*Danio rerio*) and threespine stickleback (*Gasterosteus aculeatus*).

The zebrafish has become a widely used vertebrate model in human biomedical research due to its physiological advantages, optical transparency during early development, and suitability for *in vivo* imaging and high-throughput approaches (Choi et al., 2021). Importantly, the zebrafish immune system develops in a sequential manner: the innate immune system is functional within the first day’s post-fertilization (dpf), whereas adaptive immunity appears only weeks later. This timeline in zebrafish development prior to five dpf embryos and larvae provides an opportunity to study innate immune mechanisms in isolation and to investigate the impact of toxicants on immune ontogeny. Importantly, this adheres to the 3Rs by using early-life, non-protected stages of a non-mammalian vertebrate (Franza et al., 2024).

A further aim is to develop NAMs based on high-throughput screening tools and contribute to AOPs by identifying immunotoxic pathways in the innate immune system (microglia and macrophages) using transgenic zebrafish (<120 h post-fertilization (hpf)).

To cover the requirements for environment protection and enhance the weight of evidence obtained from the zebrafish model, the use of an additional species, which is important ecologically in temperate waters and is also a widely used laboratory model will be added. The threespine stickleback is considered a “supermodel” vertebrate for ecological and evolutionary genomics in aquatic environments. A high-quality reference genome supports transcript annotation and molecular pathway analysis for functional research. Protocols for laboratory rearing of sticklebacks are well established (Blaker et al., 2022), whilst it is a validated species for overt toxicity and endocrine disruption (OECD GD 148). The sticklebacks’ natural habitat includes a wide range of environmental conditions, freshwater, brackish and marine systems. Together with adaptive radiation, i.e., the ability to adapt and evolve in a wide range of environments, and the relative ease to be kept in laboratory conditions, this offers a unique system to study the effects of chemicals. Briefly, like all fish sticklebacks develop in degree-days so the lower the temperature the slower the growth and *vice versa*. The maximum tolerated temperature is 20 °C. This offers an additional advantage to study effects of various fish pathogens with different temperature preferences. At 17 °C–18 °C the embryos hatch in 7–8 days and become protected on day 13–14, when they first show the ability to feed independently. Embryo fertilizations can employ *in vitro* fertilization (Barber and Arnott, 2000) offering an ability to study the interaction of genomic and epigenomic influences to chemical sensitivity. Supplementary Figure S1 in the Supplementary Material visualizes the model of the threespine stickleback used to investigate developmental ontogenesis and immunotoxicology.

Importantly, the species can provide an insight of immune dysfunction in the real environment (Marchand et al., 2019) and inform endocrine-related immunotoxicity (Mit et al., 2023).

Data from chemical-exposed stickleback embryos will be generated to evaluate immunosuppression and immune function during embryonic and adult stages, with the aim to evaluate long-lasting immunosuppression from early exposure. Chemical exposure will be conducted in two partner institutions and samples will be shared across laboratories for an inter-laboratory comparison.

## 4 Concluding remarks and outlook

The PARC DIT NAMs project has adopted a comprehensive approach by integrating literature-based work on DIT and human immune system development (subproject 1), that will in turn inform targeted NAMs efforts (subproject 2). This strategy aims to address the considerable insufficiencies in the regulatory risk assessment of DIT compounds. The project’s main objective is to generate fit-for-purpose methodologies that can facilitate the transition to the use of NAMs in DIT risk assessment, enabling an enhanced and more efficient strategy for the identification of chemicals with the potential to harm immune health.

To strengthen these efforts, the PARC DIT NAMs project partners will collaborate with international initiatives and other EU projects that concentrate on immunotoxicity including DIT, such as the EU projects ExpoSim and ENDOMIX, as well as the CAAT-led working group on DIT alternatives.

## Footnotes

1
https://disease-maps.io/immunedev/
