# Supplementary material for: New approach methodologies (NAMs) to support regulatory assessment of developmental immunotoxicity – a new PARC project
Source: Front Toxicol. 2026 Apr 17;8:1740390. doi: 10.3389/ftox.2026.1740390 (PMC13132502; doi:10.3389/ftox.2026.1740390)
Supplement: Supplementary file 1 [file DataSheet1.docx]

Supplementary material

**Table S1 Full Workshop Agenda and speakers**

Workshop Agenda (Day 1 March 1st 2024)

| Time (CET) | Presentation | Speaker |
| --- | --- | --- |
| 14:00 – 14:15      (15’) | Welcome  Aims of the workshop  Immunotoxicity work in PARC | Dr. Hubert Dirven, Norwegian Institute of Public Health, Norway (Chair)  Etienne Blanc, INSERM, France |
| 14:15 – 14:35      (20’) | ECHAs Research needs: Developmental Immunotoxicity | Bohumila Bichlmaier Suchanova,  ECHA, Finland |
| 14:35 – 14:55      (20’) | EFSA scientific opinion on the risk to human health related to the presence of PFAS in food: example of effects on the immune system | Prof. Tanja Schwerdtle,  BfR, Germany |
| 14:55 – 15:15      (20’) | Prenatal exposure and long-term effects on the human immune system | Dr. Berit Granum,  NIPH, Norway |
| 15:15 – 15:35      (20’) | Evaluating effects on the developing immune system: a closer look at the TDAR in the EOGRTS | Rob Vandebriel,  RIVM, The Netherlands |
| BREAK (30’) | | |
| 16:05 – 16:25      (20’) | *In vivo* approaches to assessing developmental immunotoxicity | Prof. Jamie DeWitt,  Oregon State University, USA |
| 16:30 – 17:15      (45’) | Introduction to Breakout sessions and discussions led by rapporteur | Individual rapporteurs leading 2-3 groups |
| 17:15 – 18:00 | Plenary to discuss conclusions from Breakout sessions – wrap up | Chair and all participants |

Workshop Agenda (Day 2 March 4th 2024)

| Time (CET) | Presentation | Speaker |
| --- | --- | --- |
| 14:00 – 14:15      (15’) | Welcome  Aims of the workshop  Brief summary of day 1 | Prof. Emanuela Corsini, University Milan, Italy (Chair) |
| 14:15 – 14:35      (20’) | Progress on NAMS in the CAAT DIT working group | Dr. Fenna Sillé, JHU-CAAT, USA |
| 14:35 – 14:55      (20’) | Validation of an *in vitro* method for regulatory acceptance | Silvia Casati Joint Research Centre (JRC), Italy |
| 14:55 – 15:15      (20’) | Zebrafish-based NAMs | Assoc. Prof. Anna Pistocchi, University of Milan, Italy |
| BREAK (30’) | | |
| 15:45 – 16:05      (20’) | NAMs to assess the impact of chemicals on T-cells/ Interaction between immune cells and trophoblast | Prof. Ana Zenclussen, UFZ, Germany |
| 16:05 – 16:25      (20’) | Computational approaches: Universal Immune System Simulator | Dr. Elena Crispino, University of Catania, Italy |
| 16:25 – 16:45      (20’) | Lessons learned from the DNT IVB and DIT in vitro work | Prof. Dr. Ellen Fritsche, SCAHT, Switzerland |
|  | | |
| 16:45 – 17:30      (45’) | Introduction to Breakout sessions and discussions led by rapporteur | Individual rapporteurs leading 2-3 groups |
| 17:30 – 18:00 | Discussion on next steps; sum up and meeting close | Chair and all participants |

**Table S2 Guiding questions for the breakout groups for both days of the workshop (45 min) in small participant groups to brainstorm actionable ideas.**

| **Day 1 Breakout group guiding questions** | |
| --- | --- |
| Question 1: Exploring Data Gaps in DIT Knowledge | What are the most significant gaps related to our current understanding of DIT?  Considering these gaps, what research could PARC undertake to bridge them?  What are considered the most critical developmental periods or developmental key events for disruption by chemicals? Do these have implications for DIT test development? |
| Question 2: Indications for DIT Testing | What key factors or exposures of the mother should/could trigger DIT testing?  How can we effectively use DIT testing/NAMs to identify potential DIT chemicals?  Can these tests be used to screen chemicals, set safe levels of exposure (POD), trigger for DIT cohort in EOGRTS, read across or chemical grouping? Any further uses? |
| Question 3: Regulatory Perspectives on DIT | Consider how (new) PARC's research activities could be aligned to meet regulatory needs (specific data/study design) effectively. What are the most critical needs and which ones could be addressed in new PARC projects?  What innovative approaches or adjustments might be required to bridge any existing gaps between current research practices and regulatory needs? |
| **Day 2 Breakout group guiding questions** | |
| Question 1: In vitro test battery development ​  ​ | How can we start developing a test battery for the replacement of DIT animal tests and ensure coverage of other endpoints beyond immunosuppression? What NAMs are promising (for filling data gaps, screening, chemical grouping)?  How can NAMs/in vitro testing facilitate and contribute to the development of a tiered approach in DIT testing? |
| Question 2: Criteria for NAM Acceptance and Readiness | What specific biological events should be accurately captured by NAMs to inform on the disruption caused by chemicals? ​  What kinds of evidence and methodological validation should PARC prioritise to facilitate the acceptance and standardization of NAMs? ​  ​ |
| Question 3: Enhancing Collaboration for NAM Development and Validation | How can new projects in PARC ensure that these methods/NAMs are robust, reproducible, and widely accepted by both the scientific community and regulatory agencies?  How can these collaborations be structured to accelerate the acceptance and integration of NAMs into regulatory frameworks? |


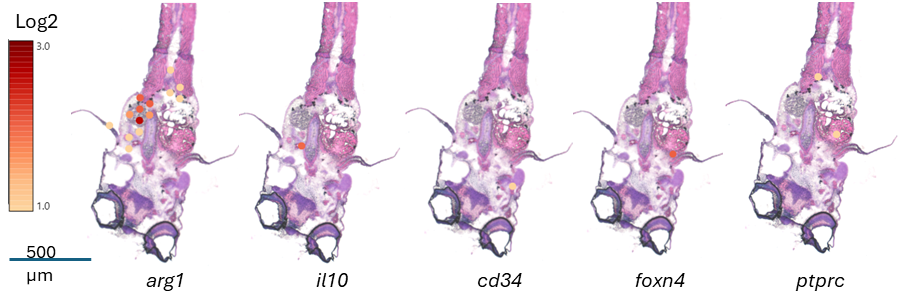


**Figure S1** **Model of the threespine stickleback used to investigate developmental ontogenesis and immunotoxicology in fish. ​**Twelve days post fertilization embryo displaying gene expression (Log2) of arginase 1 (arg1), a marker for M2-type macrophages; interleukin-10 (il10), an anti-inflammatory cytokine; cd34, a marker for haematopoietic stem cells; forkhead box protein N4 (foxn4), an essential gene for thymus organogenesis in teleosts; and protein tyrosine phosphatase receptor type C (ptprc) or CD45 antigen, expressed in haematopoietic cells, especially lymphocytes.
